# Rupture of an Unimpregnated Uterus, from a Collection of Pus in Its Cavity

**Published:** 1849-02

**Authors:** 

**Affiliations:** Naples


*Rupture of an Unimpregnated Uterus, from a Collection of Pus in its Cavity.* By Dr. Guzzo of Naples.—A woman, æt. thirty-four, liable from puberty to uterine pains and irregularities, married, but childless, came under Dr. Guzzo's care in June 1837, when he found the uterus as enlarged as at the fifth month of pregnancy, and a twelvemonth after it nearly had reached the umbilcus, occasional colourless discharges being observed. She continued to live until 1841, the tumefaction still increasing, when, after the use of a purgative, peritonitis was induced, and in a few hours she died. A large quantity of pus was found in the abdomen, and the uterus adhered to its parieties from the pubis to the umbilicus, filling up the iliac and hypochondriac regions, and was covered by the omentum. The cavity of the womb contained an enormous quantity of inodorous white pus, various irregular hypertrophic formations being developed on its inner surface. Its walls were thickened, and contained in their substance tubercular masses, varying in size from an olive to a walnut, some being crude, and others suppurating. Some of these tubercular abscesses were just on the point of opening into the cavity of the uterus, and a rupture had taken place at the posterior surface of the organ. *Brit. and For. Med. Chir. Review, from Archives Gen.*

